# The Cost‐Effectiveness of an Intervention to Preserve Independence in People With Dementia (Vs. No Intervention): A Decision‐Analytic (Markov) Model Analysis

**DOI:** 10.1002/gps.70132

**Published:** 2025-07-23

**Authors:** Luke Paterson, Rachel A. Elliott, Fofi Constantinidou, Renaud David, Piers Dawes, Eric Frison, Mark Hann, Hannah Hussain, Iracema Leroi, Antonis M. Politis, Chryssoula Thodi, Elizabeth M. Camacho, SENSE‐Cog Study Team

**Affiliations:** ^1^ Division of Population Health Health Services Research & Primary Care School of Health Sciences University of Manchester Manchester UK; ^2^ Nuffield Department of Population Health Medical Sciences Division University of Oxford Oxford UK; ^3^ Department of Psychology & Center for Applied Neuroscience University of Cyprus Nicosia Cyprus; ^4^ Nice University Hospital Université Côte d’Azur Nice France; ^5^ Centre for Hearing Research School of Health and Rehabilitation Sciences University of Queensland Brisbane Australia; ^6^ Euclid/F‐CRIN Clinical Trials Platform INSERM Institut Bergonié CHU Bordeaux CIC1401‐EC University of Bordeaux Bordeaux France; ^7^ The Office of Health Economics London UK; ^8^ Global Brain Health Institute Trinity College Dublin Dublin Ireland; ^9^ 1st Department of Psychiatry Eginition Hospital National and Kapodistrian University of Athens Athens Greece; ^10^ Department of Health Sciences European University Cyprus Nicosia Cyprus; ^11^ Institute of Population Health University of Liverpool Liverpool UK

**Keywords:** activities of daily living, cost‐effectiveness, dementia, independence

## Abstract

**Objectives:**

Interventions that enable people with dementia to retain some independence in activities of daily living (ADL) may delay transitions into residential care and offset sharp reductions in quality of life (QoL). The aim of this study was to estimate how effective a hypothetical intervention needs to be at preserving independence in home‐dwelling people with dementia, to be cost‐effective.

**Methods:**

A decision‐analytic model was constructed to compare costs and outcomes of a cohort of people with dementia in the United Kingdom and European Union over a 10‐year period. At model entry, the cohort was distributed across low, moderate, or high levels of dependence. The impact of a hypothetical intervention that preserves independence was evaluated by reducing the proportion of people entering the model with moderate and high dependence. The model included costs for the intervention and health and social care resource use. Secondary analysis included estimated costs of informal care. Health benefit was measured as quality‐adjusted life‐years (QALYs).

**Results:**

The cost of the intervention was £570/person. At this cost, an intervention that resulted in 7.5% of the sample entering the model in a lower level of dependence (compared with no intervention) was likely to be cost‐effective (£8690/QALY). An intervention costing £250/person would only need a 2.5% effect and one costing £1000/person would need to have a 10% effect to be potentially cost‐effective. Including informal care costs increased the size of the effect required for the intervention to be cost‐effective because more of the care provided at lower levels of dependence is informal.

**Conclusions:**

Preserving independence in people with dementia may be a cost‐effective way to help them live well for longer. Our results provide a guide on costs and required effects for those developing interventions to preserve independence in people with dementia.

## Key Points

- Despite recent developments, dementia currently remains without a cure, more research is needed to help people live well with dementia.
- Helping people with dementia to retain independence in activities of daily living may improve their quality of life, but there is limited evidence on the cost‐effectiveness of doing so.
- Our analysis suggests that interventions which help people with dementia retain some independence in activities of daily living may be cost‐effective.
- High‐quality evaluations of independence‐preserving interventions are needed to drive a change in evidence‐based practice.

## 1 Introduction

Dementia is a progressive neurodegenerative condition estimated to affect over 1.2 million people in England by 2050 [1]. In 2018, the annual cost of caring for people with dementia in England was estimated to be £11.7 billion and is projected to double by 2050 [1]. While some pharmacological interventions appear to temporarily stabilise or slow cognitive deterioration [2, 3], no cure currently exists. Consequently, research and policy have increasingly focussed on helping people to live well with dementia with non‐pharmacological approaches [4]. For instance, appropriate monitoring and correction of any sensory impairments (e.g. hearing or vision loss) may enable people with dementia to better engage with their surroundings [5], reduce the impact of dementia, and improve quality of life [6]. Understanding the value for money (or cost‐effectiveness) of such interventions is critical for their integration into healthcare systems.

Given finite healthcare budgets, cost‐effectiveness analysis provides evidence on whether an intervention is likely to be good value for money. This evidence supports those making decisions about which interventions to fund. It is also useful for people developing new interventions for people with dementia. It can provide guidance on how effective an intervention would have to be in order to be cost‐effective and direct research towards options that are more likely to be cost‐effective. For example, less expensive interventions require smaller effects to be deemed cost‐effective compared to more costly, complex interventions.

Dementia is a multifaceted condition that affects cognitive function, behaviour and mood, and the ability to perform activities of daily living (ADL) such as getting dressed or preparing meals [7, 8]. As dementia progresses, support from informal (e.g. family members, volunteers) and formal (i.e. paid) care partners is required to navigate daily life. Losing independence in carrying out ADL is associated with reduced quality of life (QoL) in people with dementia [9, 10] and their care partners [11], as well as increased cognitive and physical impairments [12, 13]. Loss of independence is also associated with increased health and social care resource use, including transitions into residential care [14].

Maintaining independence is a priority for people with dementia [15]. Interventions aimed at supporting independence may help to alleviate some of the pressure on care partners, and potentially delay transitions into residential care [16]. These interventions could be simple tools like portable alarms, yet interventions aimed at supporting independence are often complex [17] such as smart‐home technologies to provide reminders or to automate activities which may be implemented alongside additional one‐to‐one support [16, 18, 19]. There are many commercial devices and complex interventions designed to maintain independence; however they are seldom tested to see how well they work or if they offer good value for money. Consequently, little is known about the costs, benefits, or cost‐effectiveness of preserving independence in people with dementia. This analysis aims to estimate how effective an intervention would have to be at helping people with dementia retain independence (in carrying out ADL), to be cost‐effective.

## 2 Methods

### 2.1 The Model

**FIGURE 1:**
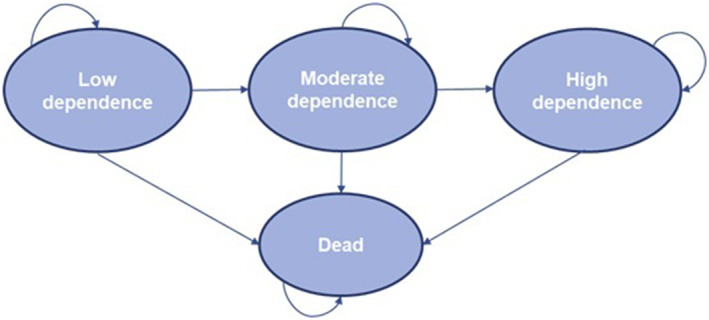
Structure of Markov model.

We developed a decision‐analytic economic model to explore the cost‐effectiveness of a hypothetical intervention that preserves independence in people with dementia compared with no intervention. The model was developed in accordance with best practice guidance [20] and is reported according to the CHEERS Checklist 2022 [21]. The model was a state‐transition Markov model (see Figure 1 for model structure). Markov models are appropriate for modelling dementia as they allow for the chronic, progressive nature of the disease in their structure [22]. A hypothetical cohort of people with dementia move through the model (from one health state to another) over time. The time horizon for the model was 10 years (which approximates a lifetime horizon in people with dementia as the average survival from diagnosis is 4–8 years [23]) and people can move between health states every 3 months (i.e. a 3‐month cycle length). The model was analysed from the perspective of the health and social care provider in England. The cost‐year for the model was 2019/20, and currency was Pounds Stirling (GBP). In the base case model, a discount rate of 3.5% was used for both costs and health effects accrued beyond the first year of the model, as recommended by national guidelines in England [24]. The model was built and analysed in Excel and reproduced in R (R version 4.3.0, http://www.r‐project.org) to validate the model. A formal health economic analysis plan was not developed for the model.

Most published Markov models of dementia involve health states that relate to level of cognitive function [22]. However, this does not take into account the progressive impacts of dementia on other domains which can also have important effects on health and wellbeing [25]. In our model the main health states are based on dependence, as captured by the Bristol Activities of Daily Living Scale (BADLS) [26], so that the progression of dementia incorporates multidomain effects including cognition, function, and behaviour. The BADLS is an objective measure of dependence in people with dementia, whereby caregivers are asked to rate the level of difficulty the person with dementia has in carrying out ADL, based on their direct observations. The BADLS is scored from 0 to 60, with a higher score indicating greater dependence (i.e. lower independence) in conducting ADL. The dependence‐based health states in our model were defined based on total BADLS score, using thresholds reported in published research: low (0–14), moderate [15, 16, 17, 18, 19, 20, 21, 22, 23, 24, 25, 26, 27, 28, 29], or high (30+) dependence [27]. There is also a health state in the model that represents when people are dead.

### 2.2 Key Data Sources

The SENSE‐Cog study is a key data source for the model [28, 29]. SENSE‐Cog was a multi‐national randomised controlled trial (RCT) of a complex intervention for people with dementia carried out in the United Kingdom (UK), Ireland, France, Cyprus and Greece. The intervention aimed to improve quality of life (QoL) in people with dementia who had hearing and/or vision impairment(s). It involved identifying and correcting the sensory impairments(s) and providing additional support and goal‐setting activities delivered by a trained therapist (described in full in the study protocol [29]). It was hypothesised that in addition to improving QoL, the intervention would also help people with dementia to retain independence in performing ADL [6]. Participants were recruited into SENSE‐Cog as dyads of people with dementia and a care partner. Care partners were required to be aged 18 years or older and an informal care partner (where providing care was not the person's primary paid role), with regular contact with the person with dementia (at least weekly). The recruitment period was from May 2018 to May 2021.

There were 177 participants randomised to receive the intervention and 177 randomised to receive care as usual (CAU). As part of the study, data were collected from participants and care partners in both trial arms to measure QoL (Dementia Quality of Life; DEM‐QoL) [30], health utility (EQ‐5D‐5L) [31], and independence in ADL (BADLS) [26] at baseline and 18‐week and 36‐week follow‐up assessments. The primary trial outcome was DEM‐QoL score measured at 36‐week after study entry. On average, the SENSE‐Cog intervention costed £570 per person to deliver (this included training sensory support therapists and therapist contact time with participants to deliver the intervention, assessment of sensory impairments, and provision of sensory augmentation devices) [28]. Although DEM‐QoL scores were significantly higher in the intervention group at 18 weeks, there was not a statistically significant difference between the intervention and control groups on any of the outcome measures at 36 weeks therefore we pooled data from the control and intervention groups to inform our model.

Another key data source was the AD2000 study, an RCT which looked at the effectiveness of donepezil in 565 English community‐resident patients with mild to moderate Alzheimer's disease [32]. In this study, the BADLS was collected several times at varying intervals over a 55‐month period, although not all participants provided data at all time points. We were granted access to the raw BADLS data from the AD2000 study. We categorised BADLS scores as low (< 15), moderate (≥ 15 – < 30), or high (≥ 30) dependence and calculated the average probability of moving from low to moderate, and from moderate to high dependence over a 3‐month period. We used this to inform the progression of dependence over time in our model.

#### 2.2.1 The Cohort

Although the SENSE‐Cog sample only included 354 people, we based our model on a cohort of 1000 people who proportionally had similar characteristics to the SENSE‐Cog sample. The SENSE‐Cog study was designed to be representative of people living with mild‐moderate dementia in Europe [28]. This included people.

- with a diagnosis of dementia in accordance with ICD‐10 (10th revision of the International Statistical Classification of Diseases and Related Health Problems) criteria for Alzheimer's disease, vascular dementia, or mixed dementia;
- with mild‐moderate dementia (defined by a Montreal Cognitive Assessment (MoCA) score ≥ 10);
- aged 60 years or older;
- living at home at study entry.

People with dementia in the SENSE‐Cog sample had a mean age of 80 years, 53% were women, the mean MoCA score was 17, and the majority (65%) had Alzheimer's disease [28]. The model cohort were assumed to be aged 80 years at model entry. No assumptions were made about the sex ratio of the cohort. The proportion of the cohort in each health state at the start of the model (i.e. model entry) is the observed distribution of the SENSE‐Cog sample at the end of that study. This was also the case for the proportion of the model cohort who started the model in the “dead” health state which was the same as the proportion of the SENSE‐Cog sample who died during the study (6%). As “dead” is an absorbing state, these people remained here for the duration of the model and did not accrue any costs or benefits therefore did not influence the model outcomes.

#### 2.2.2 Model Parameters

The building blocks of the model which represent costs, health outcomes, and dependence (model parameters) are shown in full in Table 1. Additional details of the methods used to derive parameters are reported in Supporting Information S1: (Sections 1–2). In addition to parameters derived from the SENSE‐Cog and AD2000 studies, others were identified through systematic literature searching and selected based on their relevance (e.g. based on samples from the UK or mainland Europe) and robustness (e.g. sample size, recency, use of validated outcome measures and methodologies). When people died, it was assumed that this was preceded by a non‐elective hospital admission with a corresponding cost applied. Costs were included for primary, secondary, and social care for each model cycle.

**TABLE 1:** Model parameters.

Parameter	Value	Source
Probabilities		
Distribution of cohort at model entry		
SENSE‐cog sample at end of study (*n* = 196)	Low dependence 60%	Analysis of data from the SENSE‐Cog study
	Moderate dependence 24%	
	High dependence 10%	
	Dead 6%	
Progression of dependence		
Based on BADLS scores categorised as low, moderate, or high	Probability of moving	Secondary analysis of data from the AD2000 study
	Low→moderate dependence in 3 months: 0.15	
	Probability of moving	
	Moderate→high dependence in 3 months: 0.07	
Probability of death		
Probability of death was derived from probability of survival reported in the source study	Low dependence: 0.02	Derived from Joling et al. [36] (See also Supporting Information S1: [Table 3])
	Moderate dependence: 0.038	
	High dependence: 0.05	
Costs		
Intervention		
Cost of SENSE‐cog intervention	Per person cost of SENSE‐Cog intervention: £570	SENSE‐cog trial sample [28]
Health and social care		
Costs of health and social care over 3 months (derived from annual costs)	Low dependence: £1738	Wittenberg et al. [14]
	Moderate dependence: £3239	
	High dependence: £8510	
Death—average cost of a non‐elective hospital admission	£3519	National schedule of reference costs 2019/20 [37]
Utility values		
Utility values at model entry derived from EQ‐5D‐5L and algorithm for England [35]	Low dependence: 0.788	Analysis of data from the SENSE‐Cog study
	Moderate dependence: 0.750	
	High dependence: 0.714	
	Dead: 0	

There is a unique challenge regarding the most appropriate method to capture quality of life (and health utility values) in people with dementia [33]. As dementia progresses, there is a divergence in outcomes derived from self‐ and proxy‐reported measures. When asked to rate the quality of life of someone who has dementia, care partners typically rate this at a lower level than the person with dementia rates themselves [34]. The measure of health benefit used in the model was quality‐adjusted life‐years (QALYs). QALYs combine quality and quantity of health into a single number (i.e. health over a specific time period). “Quality” of health is measured as utility values. In our primary analysis, we derived utility values (and QALYs) from EQ‐5D‐5L data collected as part of the SENSE‐Cog study, using the method recommended by the National Institute of Health and Care Excellence (NICE) at the time of the analysis [24]. The EQ‐5D‐5L was completed as “interviewer‐administered self‐report” by people with dementia. There were no differences in QALYs between the participants in the intervention and control groups in the SENSE‐Cog study (reported in Supporting Information S1: Section 1 [Table 1]), therefore we pooled utility data from both groups to inform the model. In sensitivity analyses, we also explored QALYs derived from the DEMQoL (self‐reported) and DEMQoL‐proxy (completed by the care partners of people with dementia). These two instruments were specifically designed to measure quality of life in people with dementia [30].

To incorporate the effect of ageing on health over the course of the model, we calculated age‐based utility decrements from the Health Survey for England (HSE) [35] (methods described in Supporting Information S1: Section 2.2 [Table 2]).

#### 2.2.3 The Intervention

The starting point for the model was the proportion of participants in each of the health states (low dependence, moderate dependence, high dependence, or dead) at the end of the SENSE‐Cog study. The level of dependence was derived (as described above) from the BADLS captured in the final follow‐up assessment of the study. As the SENSE‐Cog intervention was not clinically effective at 36 weeks, and there was no statistically significant difference in BADLS scores between the treatment groups [28], data from both treatment arms of the study were pooled together to represent the no intervention condition in the model. In the first instance, the modelled hypothetical intervention was assumed to preserve independence by 2.5% at a sample level compared with no intervention (“sample‐level effect”). This was operationalised in the model by moving 2.5% of people from the moderate dependence health state into the low dependence health state, and then moving 2.5% of people from the high dependence health state into the moderate dependence state at the start of the model. A 2.5% sample‐level effect was assumed to be a realistic intervention effect in real‐world settings based on available evidence [17]. The distribution of people at the start of the model was the only difference between the no intervention condition and those receiving the hypothetical intervention, subsequent transition probabilities were assumed to be the same for both groups. This conceptualises the effect of the intervention as preserving independence until the end of the trial period (i.e. 36 weeks as in the SENSE‐Cog study) but assumes an equivalent rate of progression of dependence over the course of the model (i.e. after the hypothetical intervention has been withdrawn). Alternative sample‐level effects were then explored: 5%; 7.5%; and 10%.

### 2.3 Cost‐Effectiveness Analysis

Costs and QALYs were estimated over 10 years. The number of people in each health state in each model cycle was multiplied by the costs and QALYs associated with the respective health states. These cycle‐level (3‐month) costs and QALYs were added together to produce the 10‐year outcomes. The model was run for a cohort of people who received an independence‐preserving intervention to estimate costs and QALYs over 10 years. This was repeated for a cohort who did not receive an intervention. Incremental costs and QALYs were calculated as the difference between these cohorts (intervention vs. no intervention). Incremental cost‐effectiveness ratios (ICERs) were generated by dividing the incremental cost by the incremental QALYs. This shows the cost required to improve health by one QALY. In England, the cost‐effectiveness threshold (i.e. the point at which an intervention is considered cost‐effective) used by national decision‐makers is £20,000/QALY [38]. We have interpreted the ICERs generated according to this threshold.

### 2.4 Sensitivity Analysis

Key assumptions made in the base case model were explored in sensitivity analyses. The following sensitivity analyses were conducted (see Supporting Information S1: Section 3, for additional details).

- reduced time horizon of the model
- alternative measures of health utility to calculate QALYs
- inclusion of informal care costs
- sub‐group intervention effect
- alternative intervention costs

#### 2.4.1 Stakeholder Engagement

The clinical and real‐world validity of the model were maximised by inviting and incorporating input from clinical and lived experience experts (people with dementia and their informal care partners) at key stages through the model development and interpretation of results. We selected the focus of the hypothetical intervention (i.e. preserving independence) because of their input. Experts by experience told us how changes they had witnessed in independence in performing ADL were clearly observable and progressed in a stepwise and irreversible way (i.e. once the ability to carry out an activity was lost it was not regained). This is reflected in the transitions in the model.

## 3 Results

### 3.1 Base Case Model

The base case analysis assumes an intervention cost of £570/person and models costs and QALYs over a 10‐year period with a 3.5% discount rate. The results of the base‐case model are reported in the top half of Table 2. In this model, a sample‐level effect of 7.5% would be required for the hypothetical intervention to be cost‐effective (i.e. less than £20,000/QALY) compared with no intervention. In other words, 7.5% of the sample would need to enter the model at a lower level of dependence compared with no intervention. An intervention with a sample‐level effect of 10% would be cost‐saving and health‐improving and would therefore dominate no intervention.

**TABLE 2:** Costs, QALYs, and ICERs over 10 years (and 5 years) comparing a hypothetical intervention to no intervention.

Sample‐level effect^a^ (% of sample entering model at a lower level of dependence)	Cost per person	Net cost	QALYs per person	Net QALYs	ICER^b^
10 year time horizon					
0% (no intervention)	£104,371	—	3.499	—	—
2.5%	£104,786	£415	3.504	0.0042	£99,800
5%	£104,631	£261	3.508	0.0083	£31,332
7.5%	£104,477	£106	3.512	0.0125	£8509
10%	£104,322	−£48	3.516	0.0167	Intervention dominates
5 year time horizon					
0% (no intervention)	£66,182	—	2.587	—	—
2.5%	£66,566	£384	2.590	0.0027	£143,470
5%	£66,380	£198	2.593	0.0054	£36,979
7.5%	£66,194	£12	2.595	0.0080	£1481
10%	£66,008	‐£174	2.598	0.0107	Intervention dominates

^a^ “Sample‐level effect” denotes the proportion of the sample assumed to be in a lower state of dependence (i.e. moderate→low; high→moderate) at the start of the model compared with no intervention. e.g., the proportion of the sample observed to have moderate dependence is reduced by 2.5% and these people are instead assumed to start the model in the low dependence health state.

^b^ Mean and net costs and QALYs are reported as rounded values whereas ICERs are calculated based on non‐rounded values.

#### 3.1.1 Sensitivity Analyses

The results of the model over a 5‐year time horizon are reported in the bottom half of Table 2. Compared with the 10‐year time horizon, the ICERs are larger over 5 years until an effect size of 7.5% is reached. However, the point at which the hypothetical intervention becomes cost‐effective (i.e. a sample‐level effect that preserves independence by 7.5%) is the same over both 5‐ and 10‐year time horizons.

Including costs for both formal care and informal care over 10 years had a large impact on cost‐effectiveness (Table 3). In this scenario, the intervention would not be cost‐effective at any of the sample‐level effects explored. The intervention would be required to preserve the independence of 12.5% of the sample at model entry in order to be cost‐effective. At this effect size the ICER is £17,754/QALY.

**TABLE 3:** Sensitivity analyses ‐ Costs, QALYs, and ICERs over 10 years comparing a hypothetical intervention to no intervention.

Sample‐level effect^a^ (% of sample entering model at a lower level of dependence)	Cost per person	Net cost	QALYs per person	Net QALYs	ICER^b^
Inclusion of informal care costs					
0% (no intervention)	£181,664	—	3.499	—	—
2.5%	£182,193	£528	3.504	0.0042	£126,934
5%	£182,151	£487	3.508	0.0083	£58,466
7.5%	£182,109	£445	3.512	0.0125	£35,644
10%	£182,068	£403	3.516	0.0167	£24,232
Sub‐group effect—intervention assumed only to move people from moderate to low dependence at model entry, no effect for high dependence					
0% (no intervention)	£104,371	—	3.499	—	—
2.5%	£104,867	£497	3.503	0.0033	£152,799
5%	£104,794	£423	3.506	0.0065	£65,126
7.5%	£104,721	£350	3.509	0.0098	£35,902
10%	£104,647	£277	3.512	0.0130	£21,290

^a^ “Sample‐level effect” denotes the proportion of the sample assumed to be in a lower state of dependence (i.e. moderate→low; high→moderate) at the start of the model compared with no intervention. For example, the proportion of the sample observed to have moderate dependence is reduced by 2.5% and these people are instead assumed to start the model in the low dependence health state.

^b^ Mean and net costs and QALYs are reported as rounded values whereas ICERs are calculated based on non‐rounded values.

In the base case model, the same intervention effect was assumed for people entering the model at moderate or high dependence. When it was assumed that the intervention only had an impact on people who would otherwise have entered the model with moderate independence the intervention was no longer cost‐effective at the sample‐level effects explored (Table 3). The intervention would be required to preserve the independence of 12.5% of people with moderate dependence at model entry in order to be cost‐effective. At this effect size the ICER is £12,523/QALY.

In the base case model, an intervention cost of £570 is assumed. The results based on alternative intervention costs are reported in Table 4. For an intervention costing £100 per person, the intervention would dominate no intervention for all the sample‐level effect that preserves independence explored. In fact, an intervention costing £100 per person would only require a sample‐level effect that preserves independence by approximately 1.25% to be considered cost‐effective. At a cost of £250, the hypothetical intervention would dominate no intervention at a sample‐level effect of 5%. As the cost of the intervention increases to £1000 per person, the intervention would no longer be cost‐effective compared to no intervention at the sample‐level effects explored. At this cost, the intervention would be required to preserve the independence of 12.5% of the sample at model entry in order to be cost‐effective. At this effect size and an intervention cost of £1000, the ICER is £11,143/QALY. An intervention costing £2500 per person, would be required to preserve the independence of 27.5% of the sample at model entry to be cost‐effective (resulting in an ICER of £17,834/QALY).

**TABLE 4:** Sensitivity analysis (intervention cost) ‐ ICERs over 10 years comparing a hypothetical intervention to no intervention.

Sample‐level effect (% of sample entering model at a lower level of dependence)	Intervention cost				
	£100	£250	£570 (base case)	£1000	£2500
	ICER (vs. no intervention)				
2.5%	Dominates	£22,924	£99,800	£203,103	£563,460
5%	Dominates	Dominates	£31,332	£82,983	£263,162
7.5%	Dominates	Dominates	£8509	£42,944	£163,063
10%	Dominates	Dominates	Dominates	£22,924	£113,013

*Note:* Green cells indicate where the intervention dominates no intervention (lower costs, higher QALYs) or where costs are higher but would still be considered cost‐effective (ICER<£20,000/QALY). Yellow cells indicate potential for cost‐effectiveness (ICER between £20,000‐£30,000/QALY). Red cells indicate ICERs above £30,000/QALY and are unlikely to be cost‐effective.

The base case model used QALYs derived from the EQ‐5D‐5L and a discount rate of 3.5%. Further sensitivity analyses were performed using alternative sources of utility values and alternative discount rates. These analyses did not have a material impact on the results and are reported in full in Supporting Information S1: (Section 3 [Tables 4–6]).

## 4 Discussion

Our results indicate that preserving independence in people with dementia is potentially cost‐effective. Our findings can guide the development and costing of interventions which aim to preserve independence. This is particularly timely as recognition of the importance of identifying ways to help people live well with dementia grows. Our primary estimates suggest that an intervention which costs around £600/person and reduces the level of dependence (from moderate to low or high to moderate) for 7.5% of people with dementia would be considered cost‐effective for the NHS to provide in England (as this would equate to an ICER of less than £20,000/QALY).

Our sensitivity analyses suggest that if an intervention was administered across the board but was only effective for people with low or moderate dependence, it offers less value for money. To maximise benefit, it is important to tailor and target interventions to the needs and abilities of the people receiving them. When a broader societal perspective is considered, by including costs attributed to providing informal care for people with dementia, the ICER increases beyond the threshold of cost‐effectiveness. This means that the intervention must either cost less or have a greater impact to be cost‐effective when informal care costs are included.

Although there have been recent advances in disease modifying therapies for Alzheimer's disease (e.g. lecanemab and donanemab [3, 39]), policymakers in the UK have so far (as of October 2024) decided that these treatments are not cost‐effective (and that there is limited evidence of long‐term benefits) therefore have not recommended their use [40, 41]. This highlights the importance of continued efforts to produce effectiveness and cost‐effectiveness evidence for non‐pharmacological interventions.

There is a small but growing body of evidence relating to the cost‐effectiveness of interventions that enhance or preserve independence in people with dementia. A cost‐effectiveness modelling study from the United States compared four non‐pharmacological complex interventions with a primary aim of reducing nursing home admissions for people with dementia with usual care [17]. The interventions were: MIND (care coordination and planning), NYU Caregiver (counselling and support for caregivers), ADC (care co‐management and planning), and ADS Plus (support and training for caregivers). The MIND intervention focussed on 'maximising independence'. The interventions costed between $762 to $2571 (approximately £600 to £2000) and generated a net QALY gain ranging from 0.002 to 0.005 QALYs. All were cost‐saving from a societal perspective. The authors found that taking a health and social care perspective (i.e. excluding costs of informal care) favoured interventions which kept people with dementia in the community longer versus in nursing homes sooner. This is consistent with our findings and highlights the trade‐off between the burden falling on formal and informal caregivers.

One UK‐based study reported that an exercise programme that promotes independence (in addition to promoting activity and stability) for people with early dementia is cost‐effective [42]. Another UK‐based study compared a full package of assistive technology and telecare with a basic telecare package and found that the full package was not cost‐effective [19]. There is some indication that interventions such as exercise programmes, or cognitive, psychological, or occupational therapy‐based interventions (not specifically focussed on independence) may be cost‐effective at improving some outcomes in people with dementia [43].

### 4.1 Strengths and Limitations

Our model, like all state‐transition models, represents a simplified version of reality and makes several assumptions. We have explored the impact of varying key assumptions in sensitivity analyses. A notable assumption in the base case model was that the intervention was equally effective for people with moderate or high dependence. It is plausible that some interventions may be too complex for those who have already lost a significant amount of independence in performing ADL. This is an important consideration for cost‐effectiveness because interventions that are not appropriately tailored or targeted will have limited effectiveness.

It is challenging to robustly measure health utility in people with dementia [33]. To acknowledge this concern, we tested the sensitivity of our base‐case finding (using utility values derived from the EQ‐5D‐5L) using different instruments (DEM‐QoL [self‐reported] and DEM‐QoL‐proxy). Although the absolute utility values associated with the health states in the model were different, the required effect size for the intervention to be cost‐effective was the same, regardless of which instrument was used. This suggested that the method of deriving utility values was not driving uncertainty in our findings.

The SENSE‐Cog study cohort, whose data informed the model, all had hearing and/or visual impairments alongside dementia. A recent literature review reported that cognitive and visual impairment independently lead to a decline in ADL in older adults, whereas hearing loss (without vision loss) leads to a decline only if the hearing loss is severe or for activities involving hearing [44]. The benefit of an intervention which preserves independence by addressing sensory impairment may be underestimated by measures such as the EQ‐5D‐5L and DEMQoL because they may not be responsive to changes in hearing and vision [45, 46]. We have not accounted for this in our model.

A key limitation of our model is that it does not incorporate the health impact on informal care partners from preserving the independence of the person with dementia they are caring for. Helping someone with dementia to maintain independence in ADL will potentially have an impact on the burden and quality of life of informal care partners. Although, this is anticipated to be a complex relationship. For example, enabling someone with dementia to continue dressing themselves for longer means that the burden of this does not fall on the care partner. However, when people with dementia move from their homes into residential care, the majority of the care burden switches from informal to formal care partners [14]. While there are likely to be fewer demands on informal care partners in this situation (which may increase their quality of life), there may also be negative emotions (e.g. guilt) [47, 48]. In a sensitivity analysis we have included estimated societal costs for different degrees of informal care provided but this does not capture the effects on care partners' health, wellbeing, or quality of life.

A notable assumption of our analysis was that the intervention effect is only present at model entry (i.e. reducing the proportion of people entering the model in high and moderate dependence) and there are no longer‐term effects beyond this. We opted for this approach so that the benefits of the intervention are estimated conservatively. If an intervention had a longer‐lasting effect on preserving independence, this would potentially represent better value for money and require a smaller effect size to be cost‐effective. A limitation of our analysis is that we have not explored this in sensitivity analyses.

The costs of health and social care included in the model were estimated by world‐leading researchers on the burden of dementia using robust methods [14]. The source study reported costs by level of cognitive impairment (low, moderate, or severe) whereas our health states were based on level of dependence and so we used cognitive impairment as a proxy for the level of dependence in our model (i.e. costs for low impairment used for the low dependence health state). While there are likely to be differences in the relationship between health and social care costs with cognitive impairment and dependence, we used this approach based on the robustness of the source study and the lack of published costs by level of dependence. A similar approach has been used previously in work by others [49].

### 4.2 Implications

The evidence reported here can shape the research agenda in two main ways. Firstly, we have highlighted that preserving the independence of people with dementia is potentially cost‐effective and represents a promising direction for future intervention development work. Secondly, we have provided a guide for people developing interventions for people with dementia. Our findings can be used as an approximate estimate of the level of effect required for an intervention to be cost‐effective, given the cost of the intervention.

By exploring both the health and social care provider and the societal perspectives in our analysis, we have provided evidence that can be used by either side of the ongoing debate surrounding which perspective is appropriate for cost‐effectiveness analyses [50]. From the health and social care perspective, preserving independence may be a good investment if it delays people from entering residential care. However, the picture is more complex from a societal perspective. In England, NICE recommend that intervention effects on family members should be considered in cost‐effectiveness analysis where appropriate [24]. This work demonstrates how the perspective of the cost‐effective analysis (i.e. whether or not informal care costs are incorporated) can determine whether or not an intervention is considered to be cost‐effective. Particularly in the context of dementia, interventions can have impacts on both costs (i.e. time spent providing care) and health benefits (i.e. quality of life) for family members (or others) providing informal care. Further research and advocacy are needed to identify the most robust way of incorporating this in future cost‐effectiveness analyses.

There are many different types of intervention that could help people with dementia to retain independence, and they are likely to be effective for different sub‐groups. One example would be using smart speakers [51], these could provide prompts and reminders for people to encourage them to do activities of daily living. This may be more suited to people at the earlier stages of dementia who are still able to do these activities for themselves but may not always remember to do them. Whereas another example might be music therapy that can help with sleep and improve daytime alertness [52] which may then enable people with dementia to better manage their day‐to‐day activities. However, this may be less beneficial for someone with profound hearing loss or someone who does not have disrupted sleep.

We have designed and reported our base case analysis according to the recommendations of decision‐makers in England [24]. This includes the perspective of the analysis (health and social care provider), the method for estimating QALYs (derived from the EQ‐5D‐5L), and the discount rate (3.5% for both costs and QALYs). However, we have also considered how this evidence could inform decision‐making in other settings and have reported results from an alternative perspective, over a shorter time horizon (5 years), and applying alternative discount rates (reported in Supporting Information S1: Section 3).

## 5 Conclusion

Our analysis demonstrates that preserving independence in people with dementia is a potentially promising avenue to be further explored when developing interventions to help people live well with dementia. Our results have provided a guide on costs and effect sizes than can help those working in this area to incorporate cost‐effectiveness considerations as an integral part of the intervention development process. We have highlighted the need to consider targeting and tailoring interventions to potentially improve value for money. This work has also contributed to the ongoing debate around the consideration of informal care costs in cost‐effectiveness analyses due to its impact on our results. Further work is needed to explore the impact of preserving independence on the quality of life of people providing informal care to people with dementia.

## Author Contributions

E.M.C. designed the analysis. E.M.C. and L.P. conducted the analysis and co‐wrote the manuscript. I.L., E.F., and P.D. conceived and designed the SENSE‐Cog study. All authors provided critical input on the manuscript and approved the final draft.

## Conflicts of Interest

The authors declare no conflicts of interest.

## Supporting information

Supporting Information S1

## Data Availability

The data that support the findings of this study are available from the corresponding author upon reasonable request.
