# Supplementary material for: The Cost‐Effectiveness of an Intervention to Preserve Independence in People With Dementia (Vs. No Intervention): A Decision‐Analytic (Markov) Model Analysis
Source: Int J Geriatr Psychiatry. 2025 Jul 23;40(7):e70132. doi: 10.1002/gps.70132 (PMC12286906; doi:10.1002/gps.70132)
Supplement: Supplementary file 1 — Supporting Information S1 [file GPS-40-e70132-s001.docx]

**SUPPLEMENTARY MATERIAL**

**The cost-effectiveness of an intervention to preserve independence in people with dementia (versus no intervention): a decision-analytic (Markov) model analysis**

# **Utility and quality-adjusted life-years (QALYs) in the SENSE-Cog sample**

Data to derive utility values were captured using 3 measures in the SENSE-Cog study, completed at baseline, 18- and 36-weeks. Two of the measures were completed by the person with dementia (EQ-5D-5L(The EuroQoL Group, 2024) and DEMQoL (Smith *et al.*, 2005)) and the third was completed by their informal care giver (DEMQoL proxy (Smith *et al.*, 2005)). Utility values were derived from EQ-5D-5L using the algorithm-based method recommended by the National Institute of Health and Care Excellence (NICE) at the time of the analysis (Hernández Alava *et al.*, 2022). Utility values were derived from the DEMQoL and DEMQoL-proxy using their associated value sets (Mulhern *et al.*, 2013). The number of participants with complete data at all study assessments was 188 for the DEMQoL, 197 for the DEMQoL-proxy, and 185 for the EQ-5D. Missing follow-up data were addressed using multiple imputation by chained equations, with 25 imputed datasets. QALYs were calculated using the area under the curve approach. QALYs were calculated at an individual level as mean utility multiplied by the number of years in the study. For the 36-week time horizon of this study, the mean utility value during follow-up was multiplied by 36/52 weeks. The difference in QALYs between the treatment groups was estimated using a linear regression model, controlling for participant-level covariates (age, sex, country, impairment group). The QALYs derived using the 3 measures are shown in Table S1 for each treatment group.

**Table S1. Mean QALYs between baseline and 36-week follow-up for the SENSE-Cog sample, by treatment allocation**

|  | **Intervention (n=126)** | **No intervention (n=125)** | **Adjusted difference in QALYs (intervention vs. no intervention)**  **[95% CI]** |
| --- | --- | --- | --- |
|  | **Unadjusted mean QALYs [95% CI]** | |  |
| **Utility measure: EQ-5D-5L** | 0.531  [0.507, 0.556] | 0.521  [0.493, 0.549] | 0.0090  [-0.027, 0.045] |
| **Utility measure: DEMQoL** | 0.573  [0.553, 0.594] | 0.575  [0.556, 0.594] | 0.0010  [-0.027, 0.029] |
| **Utility measure: DEMQoL-proxy** | 0.505  [0.487, 0.522] | 0.510  [0.496, 0.523] | -0.0047  [-0.027, 0.018] |

# **Derivation of model parameters – additional methods**

## *Costs*

The costs associated with each dependence health state were estimated from a published costing study by Wittenberg et al (Wittenberg *et al.*, 2019). The study provided the average cost of care (itemised into primary, secondary, social, and unpaid care cost categories) for people with dementia in England based on severity levels determined by the Mini-Mental State Examination (MMSE) (Folstein *et al.*, 1975). The costs reported were inflated to 2019/20 using recommended inflation indices for healthcare (NHS Pay and Prices Index (Curtis and Burns, 2020)). The source paper reported costs for people with mild dementia with and without “care needs” – the weighted mean of these groups was used to estimate the cost for the mild dependence health state in the model (Wittenberg *et al.*, 2019). Similarly, for the high dependence health state, a weighted mean cost for people with severe dementia (based on the proportion of people with dementia living in the community or residential care) was used.

## *Utility values and QALYs*

Participants who had not withdrawn by the end of the SENSE-Cog study were grouped according to their BADLS score (low, moderate, or high dependence). The mean utility value (based on the EQ-5D-5L completed at the end of the study) was calculated for each deprivation group. These values were attached to the relevant health states for the first year of the model (year 0). Beyond the first year, we applied an age-based utility decrement. The decrement was derived based on data from the Health Survey for England 2014 (Hernández Alava *et al.*, 2022). As shown in Table S2, we calculated the percentage change in the mean utility value for ages 80 to 90. We applied each decrement for the corresponding year of the model for all health states (other than “dead”).

**Table S2. Age-based Utility Decrement Rate by Model Years**

| **Age** | **General population utility* (midpoint of male and female value)** | **Year of model** | **Age Decrement Rate (% change from previous year)** |
| --- | --- | --- | --- |
| 80 | 0.7540 | 0 | - |
| 81 | 0.7491 | 1 | 0.652 |
| 82 | 0.7441 | 2 | 0.665 |
| 83 | 0.7391 | 3 | 0.679 |
| 84 | 0.7339 | 4 | 0.693 |
| 85 | 0.7288 | 5 | 0.707 |
| 86 | 0.7235 | 6 | 0.722 |
| 87 | 0.7182 | 7 | 0.736 |
| 88 | 0.7128 | 8 | 0.750 |
| 89 | 0.7073 | 9 | 0.765 |
| 90 | 0.7018 | 10 | 0.779 |
| *From the Health Survey England (HSE) 2014 (Hernández Alava *et al.*, 2022) | | | |

## *Probability of death*

Probability of death was derived as the inverse of the probability of survival and converted to 3-month probabilities of death to align with the cycle length of the model. The source study, published by Joling et al, was based on a sample of 9230 people with dementia in The Netherlands with the same mean age as the SENSE-Cog sample (Joling *et al.*, 2020). In the SENSE-Cog sample the median number of years since diagnosis at the end of the study was 1.7. We extracted information on survival beyond 2 years was from the source study to inform the model (shown in Table S2).

**Table S3. Data extracted from Joling et al (Joling *et al.*, 2020) for survival from time of dementia diagnosis and derived probabilities of death**

| Time since dementia diagnosis | Probability of survival | Probability of death | Probability of death (3-months) |
| --- | --- | --- | --- |
| To 2 years | 0.83 | 0.17 | 0.02 |
| To 3 years | 0.73 | 0.27 | 0.03 |
| To 4 years | 0.61 | 0.39 | 0.03 |
| To 5 years | 0.5 | 0.5 | 0.03 |
| To 6 years | 0.4 | 0.6 | 0.04 |
| To 7 years | 0.32 | 0.68 | 0.04 |
| To 8 years | 0.25 | 0.75 | 0.04 |
| To 9 years | 0.18 | 0.82 | 0.05 |

# **Sensitivity analyses**

The following sensitivity analyses were conducted:

1. reduced time horizon of the model
2. alternative measures of health utility to calculate QALYs
3. inclusion of informal care costs
4. sub-group intervention effect
5. alternative intervention costs
6. reduced time horizon of the model

A 10-year time horizon was assumed in the base case model. To explore whether there are gains in health benefit or cost saving over a shorter time period, cost and benefits were re-estimated over a time horizon of 5 years.

1. alternative measures of health utility to calculate QALYs

In the base case model, health utility was derived from the EQ-5D-5L and corresponding mapping algorithm(Hernández Alava *et al.*, 2022). To explore whether different measures of health utility change the gains in health benefit or costs, we also used the DEM-QoL and DEM-QoL-Proxy instruments. Table S3 shows the mean utility values from the SENSE-Cog study sample, derived from each measure.

**Table S4. Mean utility values from the SENSE-Cog study for EQ-5D-5L, DEM-QoL, and DEM-QoL-Proxy by Dependency Health States**

|  | **Mean utility values** | | |
| --- | --- | --- | --- |
| **Health states** | **EQ-5D-5L [95% CI]** | **DEM-QoL [95% CI]** | **DEM-QoL-Proxy [95% CI]** |
| Low | 0.788 [0.747 - 0.829] | 0.843 [0.805 - 0.881] | 0.757 [0.729 - 0.785] |
| Moderate | 0.750 [0.657 - 0.843] | 0.841 [0.787 - 0.895] | 0.724 [0.677 - 0.772] |
| High | 0.714 [0.572 - 0.857] | 0.824 [0.717 - 0.932] | 0.671 [0.599 - 0.743] |
| Dead | 0 | 0 | 0 |

1. inclusion of informal care costs

The base case model included costs attributable to formal health and social care. To explore whether adopting a broader perspective changes the gains in health benefit or costing saving, costs associated with informal (or unpaid) care for people with dementia were included in the model. The informal care costs were derived from the same source study as the health and social care costs(Wittenberg *et al.*, 2019). The 3-month costs for the representative health states were: low £5,291; moderate £8,869; high £11,540.

1. sub-group intervention effect

The base case model assumed that the hypothetical intervention had an effect on people in both the moderate dependence group (i.e. moderate 🡪 low) and the high dependence group (i.e. high 🡪 moderate). To explore this assumption, sub-group analysis was performed by assuming the hypothetical intervention only had an effect on people who were in the moderate dependence group (i.e. moderate 🡪 low) and had no effect on people in the high dependence group.

1. alternative intervention costs

In the base case model, the hypothetical intervention was assumed to have the same cost as the SENSE-Cog intervention of £570. Alternative intervention costs that cover a range of plausible intervention types (e.g. simple or complex) were explored: £100; £250; £1,000; and £2,500.

## *Results*

**Table S5.** **Sensitivity analysis (alternative source of utility values and shorter time horizon) -** **Costs, QALYs, and ICERs over 10 and 5 years** **comparing a hypothetical intervention to no intervention****, using utility values derived from the DEM-QoL and DEM-QoL-Proxy**

| **Sample-level effect***  (% of sample entering model at a lower level of dependence) | **Cost per person** | **Net cost** | **QALYs per person** | **Net QALYs** | **ICER**** |  |
| --- | --- | --- | --- | --- | --- | --- |
| **DEM-QoL (10 years)** | | | | | |  |
| 0% (no intervention) | £104,371 | - | 3.938 | - | - |  |
| 2.5% | £104,786 | £415 | 3.943 | 0.0041 | £100,636 |  |
| 5% | £104,631 | £261 | 3.947 | 0.0083 | £31,594 |  |
| 7.5% | £104,477 | £106 | 3.951 | 0.0124 | £8,581 |  |
| 10% | £104,322 | -£48 | 3.955 | 0.0165 | dominates |  |
| **DEM-QoL-Proxy (10 years)** | | | | | |  |
| 0% (no intervention) | £104,371 | - | 3.336 | - | - |  |
| 2.5% | £104,786 | £415 | 3.340 | 0.0041 | £100,713 |  |
| 5% | £104,631 | £261 | 3.344 | 0.0082 | £31,619 |  |
| 7.5% | £104,477 | £106 | 3.348 | 0.0124 | £8,587 |  |
| 10% | £104,322 | -£48 | 3.352 | 0.0165 | dominates |  |
| **DEM-QoL (5-years)** | | | | | | |
| 0% (no intervention) | £66,182 | - | 2.893 | - | - |  |
| 2.5% | £66,566 | £384 | 2.896 | 0.0025 | £155,952 |  |
| 5% | £66,380 | £198 | 2.898 | 0.0049 | £40,196 |  |
| 7.5% | £66,194 | £12 | 2.901 | 0.0074 | £1,610 |  |
| 10% | £66,008 | -£174 | 2.903 | 0.0098 | dominates |  |
| **DEM-QoL-Proxy (5-years)** | | | | | | |
| 0% (no intervention) | £66,182 | - | 2.473 | - | - |  |
| 2.5% | £66,566 | £384 | 2.476 | 0.0027 | £142,454 |  |
| 5% | £66,380 | £198 | 2.479 | 0.0054 | £36,717 |  |
| 7.5% | £66,194 | £12 | 2.481 | 0.0081 | £1,471 |  |
| 10% | £66,008 | -£174 | 2.484 | 0.0108 | dominates |  |
| * ‘Sample-level effect’ denotes the proportion of the sample assumed to be in a lower state of dependence (i.e. moderate🡪low; high🡪moderate) at the start of the model compared with no intervention. For example, the proportion of the sample observed to have moderate dependence is reduced by 2.5% and these people are instead assumed to start the model in the low dependence health state.  **Mean and net costs and QALYs are reported as rounded values whereas ICERs are calculated based on non-rounded values. | | | | | | |

**Table S6. Sensitivity analysis (alternative discount rates) – 10-year ICERs comparing the cost-effectiveness of a hypothetical intervention to no intervention**

| **ICER cost to improve health by one QALY** | | | | | |
| --- | --- | --- | --- | --- | --- |
| **Sample-level effect***  (% of sample with independence preserved) | Discount rates | | | | |
|  | 0% | 1.5% | 3.5% (base case) | 5.5% | 7% |
| 2.5% | £96,284 | £97,783 | £99,800 | £101,839 | £103,383 |
| 5% | £30,287 | £30,731 | £31,332 | £31,946 | £32,413 |
| 7.5% | £8,288 | £8,380 | £8,509 | £8,648 | £8,757 |
| 10% | dominates | dominates | dominates | dominates | dominates |
| *ICERs are calculated based on non-rounded values as the cost to improve health by one QALY. **‘Sample-level effect’ denotes the proportion of the sample assumed to be in a lower state of dependence (i.e. moderate🡪low; high🡪moderate) at the start of the model compared with no intervention. For example, the proportion of the sample observed to have moderate dependence is reduced by 2.5% and these people are instead assumed to start the model in the low dependence health state. | | | | | |

# **The list of members of the SENSE-Cog Study Team**

**MEMBERS OF THE SENSE-COG STUDY TEAM** (*left during the study)

- **Trial Development Team (TDT):**

Harvey ABRAMS, Starkey Hearing Technologies

Nathalie CHAGHIL-BOISSIÈRE, Univ. Bordeaux, INSERM, Institut Bergonié, CHU de Bordeaux, CIC-EC 1401, Euclid/F-CRIN clinical trials platform, F-33000 Bordeaux, France,

Pavlina CHARALAMBOUs, School of Sciences, Department of Health Sciences, European University Cyprus,

Fidéline COLLIN*, Univ. Bordeaux, INSERM, Institut Bergonié, CHU de Bordeaux, CIC-EC 1401,

Euclid/FCRIN clinical trials platform, F-33000 Bordeaux, France,

Fofi CONSTANTINIDOU, Center for Applied Neuroscience & Department of Psychology, University of Cyprus, Nicosia, Cyprus

Renaud DAVID, Centre Hospitalier Universitaire de Nice

Eric FRISON, Univ. Bordeaux, INSERM, Institut Bergonié, CHU de Bordeaux, CIC-EC 1401, Euclid/F-CRIN Clinical Trials Platform, F-33000 Bordeaux, France,

Camille GILBERT*, Univ. Bordeaux, INSERM, Institut Bergonié, CHU de Bordeaux, CIC-EC 1401,

Euclid/FCRIN Clinical Trials platform, F-33000 Bordeaux, France

Mark HANN, Division of Population Health, Health Services Research & Primary Care, University of

Manchester

Catherine HELMER, Centre Hospitalier Universitaire de Bordeaux

Emma HOOPER, Division of Neuroscience and Experimental Psychology, University of Manchester and the Manchester Academic Health Sciences Centre

Francine JURY, Division of Neuroscience and Experimental Psychology, University of Manchester and the Manchester Academic Health Sciences Centre

Evangelia KONTOGIANNI, 1st Department of Psychiatry, "Eginition" Hospital, National and Kapodistrian University of Athens, Greece

Brian LAWLOR, Trinity College Dublin

Iracema LEROI, Division of Neuroscience and Experimental Psychology, University of Manchester and the Manchester Academic Health Sciences Centre

Charly MATARD*, Univ. Bordeaux, INSERM, Institut Bergonié, CHU de Bordeaux, CIC-EC 1401,

Euclid/FCRIN clinical trials platform, F-33000 Bordeaux, France,

Susana MONTECELO, Essilor International

Sarah MARIE, Essilor International

Antonis POLITIS, 1st Department of Psychiatry, "Eginition" Hospital, National and Kapodistrian University of Athens, Greece

Jemma REGAN*, Division of Neuroscience and Experimental Psychology, University of Manchester and the Manchester Academic Health Sciences Centre

David REEVES, Division of Population Health, Health Services Research & Primary Care, University of Manchester

Zoe SIMKIN, Division of Neuroscience and Experimental Psychology, University of Manchester and the Manchester Academic Health Sciences Centre

Christine SCHWIMMER, Univ. Bordeaux, INSERM, Institut Bergonié, CHU de Bordeaux, CIC-EC 1401, Euclid/F-CRIN clinical trials platform, F-33000 Bordeaux, France

Monique TERMOTE, Univ. Bordeaux, INSERM, Institut Bergonié, CHU de Bordeaux, CIC-EC 1401, Euclid/FCRIN clinical trials platform, F-33000 Bordeaux, France,

Chryssoula THODI, School of Sciences, Department of Health Sciences, European University Cyprus,

Lucas WOLSKI*, Institute of Applied Research, Development and Continuing Education, Catholic University of Applied Sciences, Freiburg, Germany

- **Trial Management Team (TMT):**

Iracema LEROI, Trial management Team Chair, Dublin, Ireland

Nathalie CHAGHIL-BOISSIÈRE, CRA, Univ. Bordeaux, INSERM, Institut Bergonié, CHU de Bordeaux, CICEC 1401, Euclid/F-CRIN clinical trials platform, F-33000 Bordeaux, France

Fidéline COLLIN*, Project coordinator, Univ. Bordeaux, INSERM, Institut Bergonié, CHU de Bordeaux, CICEC 1401, Euclid/F-CRIN clinical trials platform, F-33000 Bordeaux, France

Eric FRISON, Methodologist, Univ. Bordeaux, INSERM, Institut Bergonié, CHU de Bordeaux, CIC-EC 1401,

Euclid/ F-CRIN Clinical Trials Platform, F-33000 Bordeaux, France

Camille GILBERT*, Statistician, Univ. Bordeaux, INSERM, Institut Bergonié, CHU de Bordeaux, CIC-EC 1401, Euclid/F-CRIN clinical trials platform, F-33000 Bordeaux, France

Mark HANN, Trial statistician, Manchester UK

Julie LONGOBARDI, Project coordinator, Univ. Bordeaux, INSERM, Institut Bergonié, CHU de Bordeaux, CIC-EC 1401, Euclid/F-CRIN clinical trials platform, F-33000 Bordeaux, France

Charly MATARD*, Data manager, Univ. Bordeaux, INSERM, Institut Bergonié, CHU de Bordeaux, CIC-EC 1401, Euclid/F-CRIN clinical trials platform, F-33000 Bordeaux, France

Monique TERMOTE, Univ. Bordeaux, INSERM, Institut Bergonié, CHU de Bordeaux, CIC-EC 1401, Euclid/FCRIN clinical trials platform, F-33000 Bordeaux, France

- **Trial Steering Committee (TSC):**

Pr Sandrine ANDRIEU (Chair/Independent member)

Pr Iracema LEROI, Manchester, UK (Trial Management Team Chair)

Dr Catherine HELMER, Bordeaux, France (Trial Management Team Co-Chair)

Pr Brian LAWLOR, Dublin, Ireland (Dublin site representative)

Dr Renaud DAVID, Nice, France (Nice site representative)

Dr Antonios MOUGIAS, Athens, Greece (Athens site representative / Independent member)

Pr Savvas PAPACOSTAS, Nicosia, Cyprus (Nicosia site representative / Independent member)

Ms Lynne MACRAE, Manchester, UK (Manchester sponsor representative)

Mr Eric MONCH (Nice sponsor representative)

Pr Martina HENNESSY, Dublin, Ireland (Dublin sponsor representative)

Pr Fofi CONSTANTINIDOU, Nicosie, Cyprus (Nicosia sponsor representative)

Ms Angela ALDRIDGE, Manchester, UK (EARB Chair)

Dr Christine SCHWIMMER, Bordeaux, France (Euclid representative)

Dr Eric FRISON, Bordeaux, France (Euclid methodologist)

Mr Mark HANN, Manchester, UK (Trial statistician)

Dr Harvey ABRAMS, Florida, USA (Starkey representative, permanent Observer)

Ms Sarah MARIE, Paris, France (Essilor representative, permanent observer)

Ms Julie LONGOBARDI, Bordeaux France (Euclid, Facilitator)

- **Ethical Advisory Review Board (EARB):**

Ms Angela ALDRIDGE, Manchester, UK (Chair)

Pr Iracema LEROI, Manchester, UK (Coordinating Investigator)

Pr Brian LAWLOR, Dublin, Ireland (Dublin - Principal Investigator)

Dr Renaud DAVID, Nice, France (Nice - Principal Investigator)

Pr Antonis POLITIS, Athens, Greece (Athens - Principal Investigator/Greek local sponsor representative)

Dr Chryssoula THODI, Cyprus, Nicosia (Nicosia - Principal Investigator)

Ms Lynne MACRAE, Manchester, UK (English local sponsor representative)

Ms Hannah MCCARTHY, Dublin, Ireland (Dubliners local sponsor representative)

Mr Eric MONCH/ Mme Olga KROSELJ, Nice, France (French local sponsor representatives)

Pr Fofi CONSTANTINIDOU, Cyprus, Nicosia (Nicosia co-principal investigator) Cypriot local sponsor representative)

Invited observer: David REEVES, Manchester, UK (Trial statistician/methodologist)

Facilitator: Julie LONGOBARDI, Euclid, Bordeaux, France (Project coordinator)

- **Coordinating Unit: Euclid/ F-CRIN Clinical Trials Platform**

Nathalie CHAGHIL-BOISSIÈRE, Clinical Research Associate

Fidéline COLLIN*, Project coordinator

Eric FRISON, Methodologist

Camille GILBERT, Statistician

Julie LONGOBARDI, Project coordinator

Charly MATARD*, Data manager

Christine SCHWIMMER, Euclid Executive director

Monique TERMOTE, Data manager

Cedrick WALLET, Euclid operations manager

- **Centre for Biostatistics**

Mark HANN (Blinded statistician) Division of Population Health, Health Services Research & Primary Care, University of Manchester

David REEVES (Unblinded statistician) Division of Population Health, Health Services Research & Primary Care, University of Manchester

- **Sponsors**

Lynne MacRae, University of Manchester

Antonis Politis, University of Athens

Martina Hennessy, Trinity College Dublin

Eric Monch, University Hospital of Nice Cimiez Hospital

Fofi Constantinidou, University of Cyprus

- **Sites and Study investigators**

**Manchester**: Pr Neil PENDLETON* (PI), Pr Rachel ELLIOTT (PI), Dr Laura BROWN (Co-PI)

Study staff: Matthew BURFORD, Elisabeth CAMACHO, Anita DAVIES, Piers DAWES*, Emma HOOPER*, Ian MCKENDRICK, Dani MOUNFIELD*, Luke PATERSON, Saima SHEIKH, Zoe SIMKIN, Wai Kent YEUNG, Nick WINGATE

**Lancashire Care NHS**: Pr Worthington Mark (PI)

Study staff: Sarah ANDERTON, Polly BIDWELL, Joanna COX, Rebecca DAVIES, Alison HOLDEN, Dan PULFORD, Katie SINNOTT, Caroline WOJNARROWSKI, Nosheen ZAIDI

**Northwest Boroughs NHS**: Dr SUDHINDRA RAO Vinay (PI), Dr Ashley BALDWIN (Co-PI)

Study Staff: Joanne ADAIR, Donna CUNNINGHAM, Emma ECCLES, Anna HARDEN, Heather MORRISON, Shaheen PATEL

**Dublin**: Pr Brian LAWLOR (PI), Pr Iracema LEROI (Co-PI)

Study staff: Catriona BARRETT, JP CONNELLY, Lisa CROSBY, Trevor GRIFFIN, Emilia GRYCUK, Brendan LENNON, Shauna MCGILLEN, Catherine MOLONY, Tara NESBITT*, Rachel NILAND, Oona O’CARROLL, Sarah O’ SULLIVAN, Dominic TREPEL

**Nice**: Dr Renaud DAVID (PI), Dr Zacconi-CAUVIN (co-PI), Dr Eric ETTORE (Co-PI), Dr Aurélie MOUTON, (Co-PI), Pr Philippe ROBERT (Co-PI), Dr Guillaume SACCO (co-PI)

Study Staff: Anouk BARRET, Nathalie BESSIS, Nihed BOUGDHIRI, Anne CAVEY, Marion FERRANDEZ, Auriane GROS, Nicolas GUEVARA, Marion FERRANDEZ *, Aram KARAPETYAN, Alexandra KÖNIG, Olga KROSELJ, Justine LEMAIRE, Cindy MENEZ, Salomé MOLTER*, Magali PAYNE*, Chloé SERIGNAC, Vanina OLIVERI, Patrice VIGOT, Samir YAMANI

**Athens**: Pr Antonis POLITIS (PI)

Study staff: Giannis ARMATAS, Katerina BIZBIKI, Stamatina KABANAROU, Evgenia KATIRTZOGLOU, Dimitrios KIKIDIS, Evangelia KONTOGIANNI, Maria PASSA, Evangelia STAMOULI

**Cyprus**: Pr Chryssoula THODI (PI), Pr Fofi CONSTANDINOS (Co-PI)

Study staff: Anna Pavlina CHARALAMBOUS, Andreas CHATZIKYPRIANOU, Constantinos CHRISTODOULIDES, Charoula MATTHAIOU, Flora NIKOLAOU, Juliana PROKOPIOU, Maria

THEOCHARIDES

- **Qualitative sub study coordinators**

Ines HIMMELSBACH, Catholic University of Applied Sciences Freiburg, Freiburg, Germany

Lucas WOLSKI*, Catholic University of Applied Sciences Freiburg, Freiburg, Germany

- **Sensory Support Therapists (SST) and Research Assistants (RA) leaders**

**SST**: Emma HOOPER*, University of Cumbria, UK; Catherine MOLONY, Saint James Hospital, Ireland; Alison HOLDEN, Lancashire Care NHS, UK, successively; Charoula MATTHAIOU, University of Cyprus, Cyprus;

**RA**: Wai Kent YEUNG, University of Manchester, UK

# **CHEERS Checklist 2022** **(Husereau *et al.*, 2022)**

| Section/topic | Item No | Guidance for reporting | Reported in section |
| --- | --- | --- | --- |
| **Title** | | | |
| Title | 1 | Identify the study as an economic evaluation and specify the interventions being compared. | Title page  (page 1) |
| **Abstract** | | | |
| Abstract | 2 | Provide a structured summary that highlights context, key methods, results, and alternative analyses. | Abstract  (page 2) |
| **Introduction** | | | |
| Background and objectives | 3 | Give the context for the study, the study question, and its practical relevance for decision making in policy or practice. | Introduction  (page 3) |
| **Methods** | | | |
| Health economic analysis plan | 4 | Indicate whether a health economic analysis plan was developed and where available. | Methods (page 4) |
| Study population | 5 | Describe characteristics of the study population (such as age range, demographics, socioeconomic, or clinical characteristics). | Methods (page 5) |
| Setting and location | 6 | Provide relevant contextual information that may influence findings. | Methods (page 5) |
| Comparators | 7 | Describe the interventions or strategies being compared and why chosen. | Methods (page 7) |
| Perspective | 8 | State the perspective(s) adopted by the study and why chosen. | Methods (page 4) |
| Time horizon | 9 | State the time horizon for the study and why appropriate. | Methods (page 4) |
| Discount rate | 10 | Report the discount rate(s) and reason chosen. | Methods (page 4) |
| Selection of outcomes | 11 | Describe what outcomes were used as the measure(s) of benefit(s) and harm(s). | Methods (page 6) |
| Measurement of outcomes | 12 | Describe how outcomes used to capture benefit(s) and harm(s) were measured. | Methods (page 5) |
| Valuation of outcomes | 13 | Describe the population and methods used to measure and value outcomes. | Methods (page 5) |
| Measurement and valuation of resources and costs | 14 | Describe how costs were valued. | Methods (page 6) |
| Currency, price date, and conversion | 15 | Report the dates of the estimated resource quantities and unit costs, plus the currency and year of conversion. | Methods (page 4) |
| Rationale and description of model | 16 | If modelling is used, describe in detail and why used. Report if the model is publicly available and where it can be accessed. | Methods (page 4-8) |
| Analytics and assumptions | 17 | Describe any methods for analysing or statistically transforming data, any extrapolation methods, and approaches for validating any model used. | Methods and Supplementary Material |
| Characterising heterogeneity | 18 | Describe any methods used for estimating how the results of the study vary for subgroups. | Methods (page 8) |
| Characterising distributional effects | 19 | Describe how impacts are distributed across different individuals or adjustments made to reflect priority populations. | N/A |
| Characterising uncertainty | 20 | Describe methods to characterise any sources of uncertainty in the analysis. | Methods (page 8) |
| Approach to engagement with patients and others affected by the study | 21 | Describe any approaches to engage patients or service recipients, the general public, communities, or stakeholders (such as clinicians or payers) in the design of the study. | Methods (page 8) |
| **Results** | | | |
| Study parameters | 22 | Report all analytic inputs (such as values, ranges, references) including uncertainty or distributional assumptions. | Methods (page 7) |
| Summary of main results | 23 | Report the mean values for the main categories of costs and outcomes of interest and summarise them in the most appropriate overall measure. | Results  (page 9) |
| Effect of uncertainty | 24 | Describe how uncertainty about analytic judgments, inputs, or projections affect findings. Report the effect of choice of discount rate and time horizon, if applicable. | Results  (page 9) |
| Effect of engagement with patients and others affected by the study | 25 | Report on any difference patient/service recipient, general public, community, or stakeholder involvement made to the approach or findings of the study | Methods (page 8) |
| **Discussion** | | | |
| Study findings, limitations, generalisability, and current knowledge | 26 | Report key findings, limitations, ethical or equity considerations not captured, and how these could affect patients, policy, or practice. | Discussion  (page 11) |
| **Other relevant information** | | | |
| Source of funding | 27 | Describe how the study was funded and any role of the funder in the identification, design, conduct, and reporting of the analysis | Page 14 |
| Conflicts of interest | 28 | Report authors conflicts of interest according to journal or International Committee of Medical Journal Editors requirements. | Page 14 |

# **References**

**Curtis, L. A. and Burns, A.** (2020). *Unit Costs of Health & Social Care 2020*: PSSRU, University of Kent.

**Folstein, M. F., Folstein, S. E. and McHugh, P. R.** (1975). “Mini-mental state”: a practical method for grading the cognitive state of patients for the clinician. *Journal of psychiatric research*, 12, 189-198.

**Hernández Alava, M., Pudney, S. and Wailoo, A.** (2022). Estimating EQ-5D by Age and Sex for the UK. *NICE DSU Report*.

**Husereau, D.*, et al.*** (2022). Consolidated Health Economic Evaluation Reporting Standards 2022 (CHEERS 2022) statement: updated reporting guidance for health economic evaluations. *MDM Policy & Practice*, 7.

**Joling, K. J.*, et al.*** (2020). Time from diagnosis to institutionalization and death in people with dementia. *Alzheimer's &amp; Dementia*, 16, 662-671.

**Mulhern, B.*, et al.*** (2013). Development of DEMQOL-U and DEMQOL-PROXY-U: generation of preference-based indices from DEMQOL and DEMQOL-PROXY for use in economic evaluation. *Health Technology Assessment (Winchester, England)*, 17, v.

**Smith, S.*, et al.*** (2005). Measurement of health-related quality of life for people with dementia: development of a new instrument (DEMQOL) and an evaluation of current methodology. *Health Technology Assessment (Winchester, England)*, 9, 1-iv.

**The EuroQoL Group** (2024). EQ-5D Products.

**Wittenberg, R.*, et al.*** (2019). The costs of dementia in England. *International Journal of Geriatric Psychiatry*, 34, 1095-1103.
